# The functional roles of neural remapping in cortex

**DOI:** 10.1167/jov.20.9.6

**Published:** 2020-09-04

**Authors:** James W. Bisley, Koorosh Mirpour, Yelda Alkan

**Affiliations:** Department of Neurobiology, David Geffen School of Medicine at UCLA, Los Angeles, CA, USA; Jules Stein Eye Institute, David Geffen School of Medicine at UCLA, Los Angeles, CA, USA; Department of Psychology and the Brain Research Institute, UCLA, Los Angeles, CA, USA; Department of Neurobiology, David Geffen School of Medicine at UCLA, Los Angeles, CA, USA; Department of Neurobiology, David Geffen School of Medicine at UCLA, Los Angeles, CA, USA

**Keywords:** remapping, lateral intraparietal area, frontal eye field, visual stability, saccade, inhibition of return, inhibitory tagging

## Abstract

Remapping is a property of some cortical and subcortical neurons that update their responses around the time of an eye movement to account for the shift of stimuli on the retina due to the saccade. Physiologically, remapping is traditionally tested by briefly presenting a single stimulus around the time of the saccade and looking at the onset of the response and the locations in space to which the neuron is responsive. Here we suggest that a better way to understand the functional role of remapping is to look at the time at which the neural signal emerges when saccades are made across a stable scene. Based on data obtained using this approach, we suggest that remapping in the lateral intraparietal area is sufficient to play a role in maintaining visual stability across saccades, whereas in the frontal eye field, remapped activity carries information that affects future saccadic choices and, in a separate subset of neurons, is used to maintain a map of locations in the scene that have been previously fixated.

## What is remapping?

First described in detail by Duhamel, Colby, and Goldberg (1992), remapping is a mechanism by which neurons update their responses around the time of an eye movement to account for the shift of a stimulus on the retina due to the saccade. Such remapping has been found in subpopulations of neurons in the lateral intraparietal area (LIP; Duhamel et al., 1992; Heiser & Colby, 2006; Kusunoki & Goldberg, 2003), the frontal eye field (FEF; Joiner, Cavanaugh, & Wurtz, 2011, 2013; Umeno & Goldberg, 1997, 2001; Zirnsak, Steinmetz, Noudoost, Xu, & Moore, 2014), the superior colliculus (Churan, Guitton, & Pack, 2011, 2012a, 2012b; Walker, Fitzgibbon, & Goldberg, 1995), and in a number of visual cortical areas (Hartmann, Zirnsak, Marquis, Hamker, & Moore, 2017; Marino & Mazer, 2018; Nakamura & Colby, 2000, 2002; Neupane, Guitton, & Pack, 2016). In this review, we will focus primarily on remapping in LIP and FEF, two areas thought to be involved in the allocation of covert attention and in guiding saccadic behavior and which have been tested using tasks that better test the function of remapping rather than testing the underlying mechanisms.

**Figure 1.**
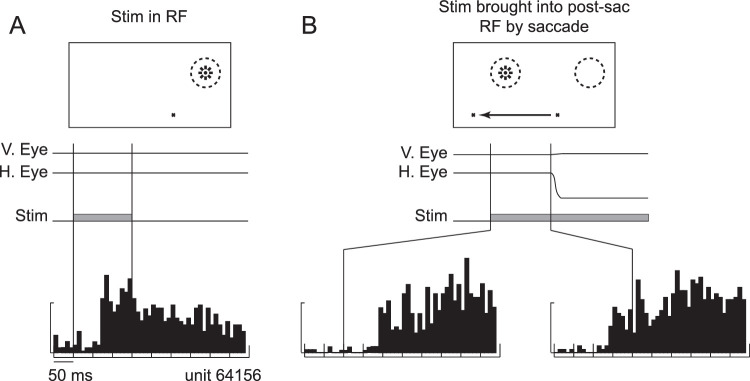
Predictive remapping of activity in a single LIP neuron. (A) The response of the neuron to a stimulus (Stim) flashed in its response field (RF), aligned by stimulus onset. (B) The response of the neuron to a stimulus brought into the postsaccadic RF by a saccade. The histogram on the left is aligned by stimulus onset, and the histogram on the right is aligned by saccade onset. V. Eye: vertical eye position. H. Eye: horizontal eye position. Modified from Duhamel, Colby, and Goldberg (1992) with permission.

We will start by clearly illustrating what we mean by the term *remapping*. Figure 1 shows a single-neuron example of this from LIP (Duhamel et al., 1992). Figure 1A shows the response of the neuron to a stimulus (asterisk in the top panel) presented for 150 ms in the neuron's response field (dashed circle) when the animal was fixating the fixation point (small cross). Following the neuron's visual latency, the neuron becomes robustly active. Figure 1B shows the response of the same neuron in a trial in which the stimulus (asterisk in the top panel) is presented in a location that will be covered by the neuron's response field (dashed circle) after the animal makes a saccade from the fixation point on the right to the fixation point on the left—we will refer to this as the “postsaccadic response field.” Before the saccade, the stimulus is not in the current response field of the neuron, which we will refer to as the “presaccadic response field.” The lower left panel of Figure 1B shows the neuronal response aligned by stimulus onset. The neuron does not begin to fire at the visual latency (as in Figure 1A), because the stimulus was not presented in the presaccadic response field, but when aligned by saccade onset (lower right panel, Figure 1B), it is clear that the neuron becomes active before the saccade even begins. At this point, the stimulus is not in the neuron's presaccadic response field, so the response cannot be driven by a response from the retina. Instead, it is thought that the activity is remapped within LIP, taking into account the vector of the saccade, such that the response from another neuron, in whose response field the stimulus was initially presented, is somehow passed (remapped) to this neuron, in whose response field the stimulus will end up after the saccade. This response is often called predictive remapping, because the neuron's response occurs earlier than could be expected from the afferent response from the retina and is predictive of how it will respond once the stimulus is in the response field.

**Figure 2.**
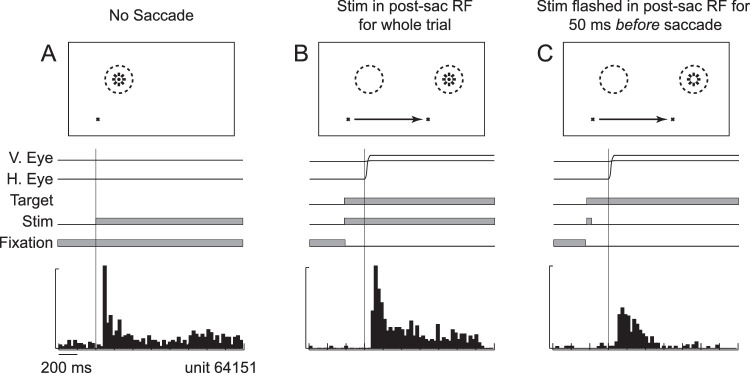
Remapping of predictive activity in a single LIP neuron. (A) The response of the neuron to a stimulus (Stim) presented in its response field (RF) during fixation, aligned by stimulus onset. (B) The response of the neuron to a stimulus brought into the postsaccadic RF by a saccade, aligned by saccade onset. (C) The response of the neuron when the location of a flashed stimulus was brought into the postsaccadic RF by a saccade, aligned by saccade onset. This response represents the remapping of the persistent activity to the flashed stimulus. The time course of the trials is shown in the middle of each panel. Modified from Duhamel, Colby, and Goldberg (1992) with permission.

Remapping is not always predictive. Because neurons in LIP and FEF often have persistent activity in response to flashed stimuli (Barash, Bracewell, Fogassi, Gnadt, & Andersen, 1991), it is possible to look for remapping at latencies longer than the visual latency. To do this, one can briefly present a stimulus in the postsaccadic response field before the eye movement, making sure that no visual signal is on the retina by the time the eye starts to move. This is illustrated in Figure 2, which shows the response of a single LIP neuron under three conditions (Duhamel et al., 1992). The first two panels show the response of the neuron to a stimulus appearing in its response field (Figure 2A) and to a stimulus being brought into its response field by a saccade (Figure 2B). Unlike the example neuron in Figure 1, this neuron did not start responding until after the saccade brought the stimulus into its response field, suggesting that it does not predictively remap. Nonetheless, Figure 2C shows that the neuron does have a remapping response. In this example, the stimulus was flashed for 50 ms in the postsaccadic response field well before the saccade was made, so by the time the saccade was made, there was nothing on the screen. The response seen is due to remapping of the persistent activity, which was generated by the neuron in whose response field the stimulus was originally flashed. Given that remapping occurs to both stimulus-evoked responses and persistent activity that is no longer driven by a stimulus, it is likely that the mechanism underlying remapping does not care about the genesis of the neuronal response; it just remaps the current activity across saccades.

Why is the presence of remapping exciting? Because it provides a possible mechanism that could aid in maintaining the perception of a stable visual world across eye movements. Each time the eye moves, there is a rapid movement with a blurring of the visual scene across the retina and the representation within visual cortex jumps from the presaccadic to the postsaccadic visual input. The former appears to be blanked out by a mechanism termed *saccadic suppression* (Burr, Morrone, & Ross, 1994). Also, there is a long history, going back to Aquilonius and Descartes in the 17th century, suggesting that the latter could be dealt with by a mechanism within the brain that can account for internally generated eye movements (for details, see Bridgeman, 2007; Wurtz, 2008). Remapping is direct evidence of an extraretinal mechanism affecting neuronal responses in a way that accounts for saccades. In the past 15 years, a number of studies have shown several components of this process, including the presence of a corollary discharge in neurons connecting the superior colliculus with FEF (Sommer & Wurtz, 2004a, 2004b) and that inactivating this discharge affects remapping responses in FEF (Sommer & Wurtz, 2006) and behavioral estimates of perceived eye position (Cavanaugh, Berman, Joiner, & Wurtz, 2016). It is not known how this signal generates remapping, but we have operated under the supposition that remapping only occurs when there is a corollary discharge signal, or a delayed relay of that signal, of a certain strength. In any case, remapping, as described above, could somehow contribute “to the construction of a continuously accurate, retinocentric representation of visual space” (Duhamel et al., 1992) by allowing the brain to account for eye movements.

## The traditional approach: Benefits and drawbacks

Studies examining remapping have typically followed a fairly standard set of procedures. A single stimulus is typically presented around the time when an eye movement is expected to be made and the physiological metrics have been whether the neuron responds and, if so, the time at which it starts responding (as illustrated in Figures 1 and 2). Using this technique, remapping has been identified in LIP (Duhamel et al., 1992; Heiser & Colby, 2006; Kusunoki & Goldberg, 2003), FEF (Joiner et al., 2013; Umeno & Goldberg, 1997, 2001), superior colliculus (Churan et al., 2012a; Walker et al., 1995), and earlier visual areas (Nakamura & Colby, 2000, 2002). A number of studies have used this technique, presenting stimuli in multiple different locations, albeit only one at a time (Churan et al., 2011, 2012b; Marino & Mazer, 2018; Neupane et al., 2016; Zirnsak et al., 2014, but see Joiner et al., 2011, in which multiple stimuli were presented at once). These studies have shown that under specific conditions, responses to flashed stimuli may show remapping toward the saccade target (Hartmann et al., 2017; Neupane et al., 2016; Zirnsak et al., 2014), although under most conditions, remapping, as illustrated in Figure 1, is seen (Hartmann et al., 2017; Neupane et al., 2016).

This standard approach provides an excellent way to probe the effects of remapping. The punctate stimulus on a plain background allows the experimenter to easily identify the onset of responses above relatively quiescent “baseline” levels and provides a sensitive metric to study the phenomenon. However, when thinking about the function of remapping in normal oculomotor behavior, there are two significant drawbacks of this technique.

**Figure 3.**
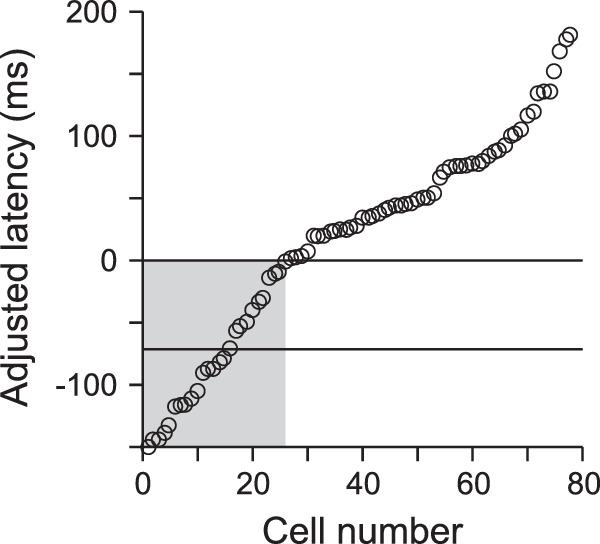
Cumulative distribution of adjusted latency of remapping in a population of FEF neurons. Adjusted latency represents the difference between the onset of the remapped response and the visual latency calculated for each neuron. Adjusted latencies less than 0 (highlighted in gray) are found in neurons that predictively remap. Adjusted latencies less than –70 (lower horizontal line) mean that the remapping response started before saccade onset. Modified from Umeno and Goldberg (1997) with permission.

First, when the timing of a traditional remapped response is examined, one finds that the onsets of remapped responses are highly variable across neurons (Kusunoki & Goldberg, 2003; Umeno & Goldberg, 1997). This is best illustrated in Figure 3, which shows the onset of the response to a stimulus briefly presented in the postsaccadic response field in a series of FEF neurons (Umeno & Goldberg, 1997). The authors present the data as “adjusted latency,” where an adjusted latency of 0 represents when the response could reach FEF from the retina, based on each neuron's visual latency. Adjusted latencies less than 0 show predictive remapping, and adjusted latencies below the lower line come from neurons in which the response began before the saccade even began (like the neuron in Figure 1). Because the stimulus was only presented before the saccade, all of the neurons in this figure display remapping, but only a subset shows predictive remapping.

The critical point to be taken from Figure 3 is that there is a huge range in adjusted latencies: up to 150 ms when looking at predictive remapping alone (grayed area). If remapping is to play a role in visual stability, then this lack of temporal stability would seem to be a substantial concern. We know that stimuli flashed around the time of a saccade are, in fact, poorly localized. Subjects either mislocalize the stimulus in a direction parallel to the saccade (Dassonville, Schlag, & Schlag-Rey, 1992; Honda, 1989, 1991) or mislocalize stimuli toward the goal of the saccade (Honda, 1993; Ross, Morrone, & Burr, 1997). Indeed, it is generally thought that these mislocalizations are due to the remapping mechanism (Cicchini, Binda, Burr, & Morrone, 2013). Given that flashed stimuli do not lead to stable visual percepts and that flashed stimuli do not lead to temporally stable responses, we would argue that, even though flashed stimuli are useful in studying the mechanisms of remapping, they might not be the best probes for testing the function of remapping in everyday behavior, particularly if it is involved in visual stability.

The second limitation of interpreting traditional remapping studies is that they look at when the remapping response begins. This time effectively represents the neural latency, yet neural latency is typically not considered an encoding metric in the areas in which these neurons are found. Instead, it is the level of response of the neurons that tends to encode features of interest (Bisley & Goldberg, 2003; Gottlieb, Kusunoki, & Goldberg, 1998; Platt & Glimcher, 1997; Thompson, Hanes, Bichot, & Schall, 1996) or drive movements (Bruce & Goldberg, 1985; Mazzoni, Bracewell, Barash, & Andersen, 1996).

Given these two issues, we propose that to understand the function of remapping, it is critical to see when an informative signal in response to stable visual stimuli emerges in the neuronal activity. This requires a task with stable visual stimuli that elicit at least two different magnitudes of responses and in which animals can make at least two saccades. Below we will describe data from several tasks that fulfill these criteria, including the stable array task of Gottlieb and colleagues (Gottlieb et al., 1998; Kusunoki, Gottlieb, & Goldberg, 2000), a free viewing visual search task (Glaser, Wood, Lawlor, Segraves, & Kording, 2020; Phillips & Segraves, 2010), and a free viewing visual foraging task (Mirpour, Arcizet, Ong, & Bisley, 2009; Mirpour & Bisley, 2012, 2016). To the best of our knowledge, the only other study that has attempted to look at the remapping of response magnitude in LIP or FEF used brief presentations (Subramanian & Colby, 2014), so it is difficult to interpret their data in terms of visual stability.

As noted above, a number of studies have identified remapping in early and mid-level visual areas (Inaba & Kawano, 2014; Marino & Mazer, 2018; Nakamura & Colby, 2002; Neupane et al., 2016; Yao, Treue, & Krishna, 2016, but see Ong & Bisley, 2011). While none of these have used naturalistic tasks, one did attempt to see whether information about the stimulus was remapped; this was done in the middle temporal area (MT) (Yao et al., 2016). The authors found that neither information about the stimulus nor attentional modulation was reliably remapped when the stimulus remained on the screen across the saccade, but they found that a memory trace was remapped and this was modulated by attention. These data suggest that the remapping in early visual areas is likely driven by top-down effects of remapping in areas such as LIP or FEF, which are known to modulate responses in early visual areas (Herrington & Assad, 2010; Moore & Armstrong, 2003).

We are also aware of the rich literature examining behavioral effects thought to be due to remapping mechanisms. These studies inform us about the global effects of remapping under the specific conditions tested and, when combined with imaging data, provide insights into the mechanisms underlying remapping. However, behavioral results, which illustrate the overall effects of remapping, do not allow us to identify specific roles of remapping in single areas, and functional magnetic resonance imaging and electroencephalogram studies are still far removed from the single-neuron level. As such, we focus on interpreting neuronal responses seen in naturalistic behavioral tasks to understand the functional roles of remapping in neurons in LIP and FEF.

## LIP as a priority map

Before describing the functional role of remapping in LIP, it is important to understand the role we think LIP plays in guiding attention. We (Bisley & Goldberg, 2010; Bisley & Mirpour, 2019; Zelinsky & Bisley, 2015) and others (Gottlieb, Balan, Oristaglio, & Suzuki, 2009) have described LIP as a priority map that is used to guide eye movements (Ipata, Gee, Goldberg, & Bisley, 2006; Mazzoni et al., 1996; Snyder, Batista, & Andersen, 1998) and covert visual attention (Bisley & Goldberg, 2003; Herrington & Assad, 2009; Ibos & Freedman, 2016). In this view, neuronal activity correlates with priority: a measure of how important the location or stimulus within the response field is. We have hypothesized that covert attention is allocated to the peak of the priority map (Bisley & Goldberg, 2006) when an unambiguous peak is present (Arcizet, Mirpour, Foster, & Bisley, 2018) and that eye movements will be made to the peak of the map when the subject chooses to move their eyes (Ipata et al., 2006). Priority is driven by low-level salience (Arcizet, Mirpour, & Bisley, 2011) as well as a host of top-down factors. Here we use the term *top-down* to refer to any factor that is not bottom-up salience. This can include reward expectation (Dorris & Glimcher, 2004; Louie & Glimcher, 2010; Platt & Glimcher, 1999; Sugrue, Corrado, & Newsome, 2004), the similarity of a stimulus to a defined target shape (Ipata, Gee, Bisley, & Goldberg, 2009; Ipata et al., 2006; Mirpour et al., 2009; Ogawa & Komatsu, 2009; Ong, Mirpour, & Bisley, 2017; Thomas & Pare, 2007) or category (Freedman & Assad, 2006; Swaminathan & Freedman, 2012), inhibition of return (Mirpour et al., 2009), behavioral state (Zhang, Wang, & Goldberg, 2014), and gains in information not directly linked to a reward (Foley, Kelly, Mhatre, Lopes, & Gottlieb, 2017; Horan, Daddaoua, & Gottlieb, 2019). This hypothesis explains why LIP activity correlates with seemingly more complex factors, such as decision-making variables (Ibos & Freedman, 2017; Janssen & Shadlen, 2005; Kiani & Shadlen, 2009; Roitman & Shadlen, 2002; Shadlen & Newsome, 2001; Yang & Shadlen, 2007): When a choice target is placed in an LIP neuron's response field, the response represents the behavioral relevance of that stimulus under the demands of that task, and this fluctuates as the decision of whether to move to that target or not fluctuates (Christopoulos, Kagan, & Andersen, 2018; Shushruth, Mazurek, & Shadlen, 2018).

In 2010, Cavanagh and colleagues proposed that remapping could lead to visual stability by shifting attentional pointers (Cavanagh, Hunt, Afraz, & Rolfs, 2010). Given that covert attention is allocated to the location in LIP with the greatest activity, one could describe that peak as an attentional pointer. Thus, their hypothesis could be instantiated by remapping within LIP, but for this to be behaviorally relevant, the remapping should occur in a more constrained temporal window than the 150+ ms seen previously. Below we describe results that show this occurs: activity in LIP shifts from the presaccadic representation of the visual scene to the postsaccadic representation within 25 ms after each saccade.

## Remapping attentional priority in LIP

**Figure 4.**
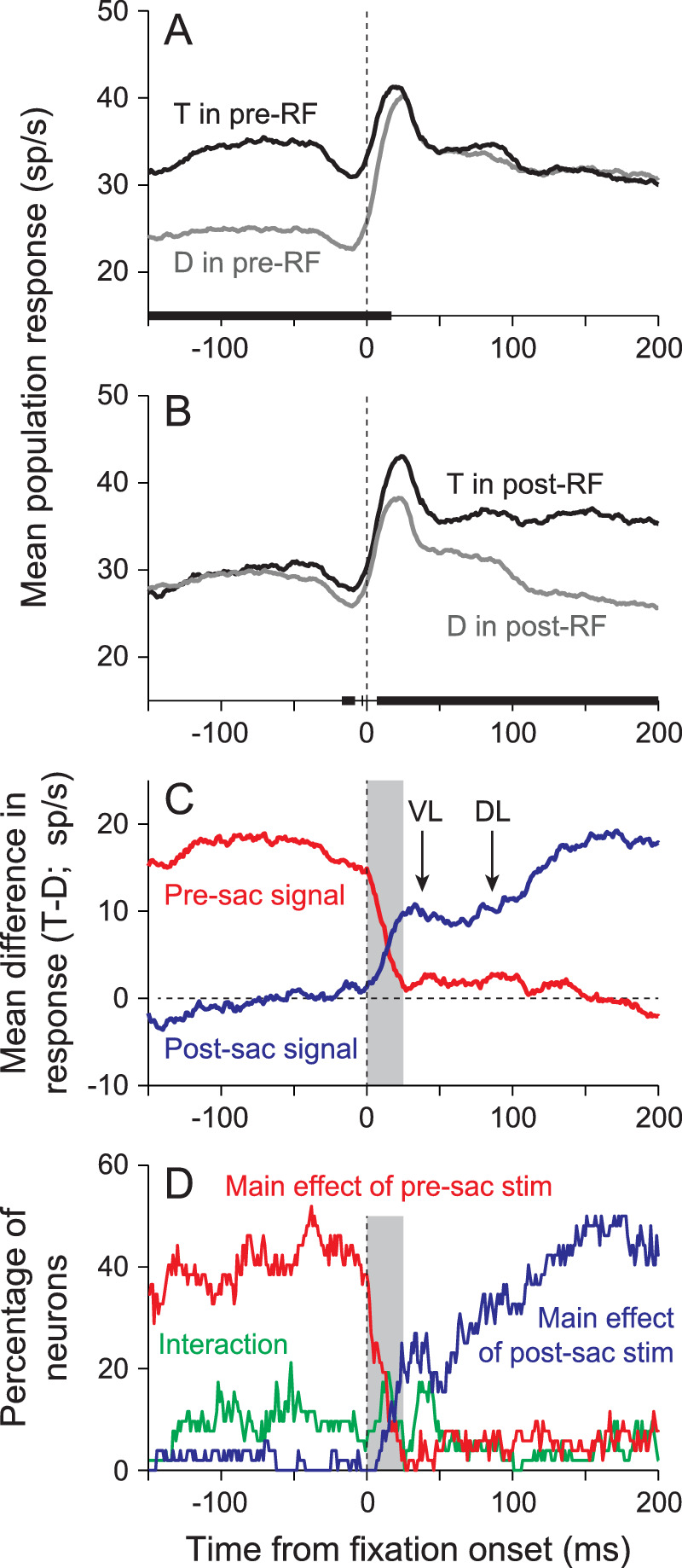
Remapping of signal in a population of LIP neurons. (A, B) The mean population response, aligned by fixation onset, is plotted as a function of the identity of the stimulus in the presaccadic response field (pre-RF; A) and as a function of the identity of the stimulus in the postsaccadic response field (post-RF; B). Black lines show responses to targets (T), and gray lines show responses to distractors (D). The solid black line along the x-axis shows times at which the two traces were significantly different (*p* < 0.01, Wilcoxon signed rank tests). (C) The difference in response to a target and distractor (T-D), aligned by fixation onset, is plotted as a function of the identity of the stimulus in the presaccadic response field (red) and the postsaccadic response field (blue). We refer to this difference in response as the neural signal. VL = mean visual latency for this population of neurons to array onset; DL = discrimination latency (i.e., the time at which the population response significantly differs when a target or distractor appears in the neurons’ response fields). (D) The percentage of neurons that showed significant differences in response based on stimulus identity (*p* < 0.01, analysis of variance), aligned by fixation onset. Red trace shows the percentage of neurons in which there was a significant difference in response to a target and distractor in the presaccadic response field, blue traces indicate the percentage of neurons in which there was a significant difference in response to a target and distractor in the postsaccadic response field, and the green traces show the percentage of neurons that had a significant interaction. Shaded gray region shows the first 25 ms after fixation onset. Modified from Mirpour and Bisley (2016) with permission.

To show the timing of remapping in LIP, we present data from a free-viewing visual foraging task (Mirpour et al., 2009). In this task, subjects are presented with an array of stimuli that remains on the screen for the duration of the trial. These typically include five identical distractors that never give a reward and five identical potential targets, one of which will be rewarded if it is fixated for 500 ms. This leads to a form of visual search in which the subjects visually forage among the stimuli, typically looking from target to target, waiting at each to see if they get the reward and then moving on. Figures 4A and 4B show the mean population response of 52 LIP neurons recorded in the visual foraging task (Mirpour & Bisley, 2016), aligned by fixation onset (i.e., the end of the saccade) and sorted by the identity of the stimulus (target or distractor) in the neuron's presaccadic response field (Figure 4A) or postsaccadic response field (Figure 4B). The blue trace in the Figure 4C shows the mean difference in response between the two traces in Figure 4B: This represents the strength of the signal of the postsaccadic response, and we will refer to it as the postsaccadic signal. Prior to fixation onset, the postsaccadic signal is close to zero, but shortly after the saccade ends, the mean difference ramps up to a plateau across a 25-ms period. It then stays at that approximate level for another 70 to 80 ms before ramping up again. The two arrows in the figure show the mean visual latency (VL) for these neurons (i.e., the time it takes from when a stimulus is presented in the response field to when the LIP neuron starts responding) and the population discrimination latency (DL), which is the time at which the activity from this population of neurons starts discriminating between the response to a target and the response to a distractor following array onset. The red trace in this figure shows the difference between the two traces in Figure 4A (i.e., the presaccadic signal), sorted based on what was in the response field before the saccade. Until fixation onset, the presaccadic signal is relatively strong, and this drops to around zero within about 25 ms. Figure 4D shows the same data set, but instead of showing the difference in response, it shows the percentage of neurons in each time bin that had a significant main effect of stimulus identity in the presaccadic response field (red), a main effect of stimulus identity in the postsaccadic response field (blue), or a significant interaction between the two (all *p* < 0.01, analysis of variance).

In these data, the postsaccadic signal plateaued out by the time of the visual latency (VL arrow in Figure 4C) and well before the population discrimination latency (DL arrow in Figure 4C). This means that the ramping up of this signal cannot be coming from the retina and thus is due to predictive remapping. Figure 4D shows that this is driven by a rapid increase in the number of neurons encoding this signal shortly after fixation onset. So unlike the traditional measures of remapping, which have found that neurons can start responding well before saccade onset, these results show that a significant signal does not occur until just after the saccade ends. This result highlights the importance of looking for a signal (i.e., the difference in response to different stimuli), as opposed to just looking to see when neurons start responding. Based on previous studies, it is likely that a subset of the neurons here would start their response before saccade onset when tested conventionally, but none show any significant signal until after the saccade. Indeed, more than 20% of all LIP neurons begin to show a significant signal during this window (shading in Figure 4D). At the same time, the percentage of neurons that significantly encoded the presaccadic signal drops from 40% to 50% down to levels expected by chance. In fact, there is only a 25-ms window (shading in Figure 4C) in which the population response does not clearly represent the pre- or postsaccadic scene.

As noted above, the postsaccadic signal starts to rise again at a time similar to the population discrimination latency (DL arrow in Figure 4C). This appears to be due to two factors. First, the number of neurons that significantly encodes the postsaccadic response starts increasing at this time (Figure 4D). These additional neurons are ones that do not predictively remap and are responding to the stimulus appearing in their response field. Second, the signal in the neurons that displayed remapping becomes more robust. In other words, the difference in responses to targets and distractors becomes greater starting at this time. These two factors are likely to have the same genesis: the retina. Both of these begin at the time that the signal differentiating targets from distractors would arrive in LIP after array onset.

**Figure 5.**
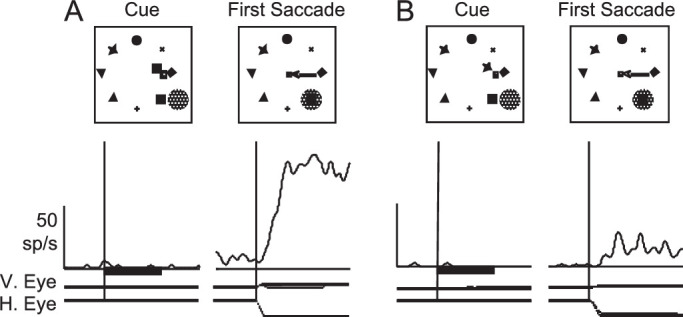
Response of a single LIP neuron in the stable array task. (A, B) During the cue period (left panels, aligned by cue onset), the animal fixated the stimulus on the right side of the array and was presented with a cue, indicating which stimulus would give the animal a reward. Animals made the first saccade (right panels, aligned by saccade onset) to the center of the array, which brought either the cued stimulus (A) or an uncued stimulus (B) into the response field (hatched circle). Modified from Kusunoki, Gottlieb, and Goldberg (2000) with permission.

A key reason for why these results are so clear is that the remapping occurred for every saccade. Neither the direction nor the length of the saccade affected the remapping (Mirpour & Bisley, 2012), and it occurred whether a target or distractor was in the pre- and/or postsaccadic response field (Mirpour & Bisley, 2016). Functionally, this means that the entire representation of visual space in LIP is remapped after each saccade. Consistent with this finding, a close examination of data from the stable array task (Figure 5) shows a similar result. In this experiment, subjects were presented with a stable array of stimuli for the entire block. On individual trials, the subjects were shown which stimulus would be rewarded. They then made a saccade to bring their eye to the center of the array (the “first saccade” in Figure 5), which brought one of the stimuli into the LIP neurons’ response field, after which they then had to make a saccade to the rewarded stimulus. Figure 5 shows the response of a single neuron when the cued (i.e., behaviorally relevant) stimulus was brought into the response field by the saccade (Figure 5A) or when an uncued (i.e., a behaviorally irrelevant stable stimulus) was brought into the response field (Figure 5B). While the large response to the behaviorally relevant stimulus is obvious, the irrelevant stable stimulus (with low attentional priority) still elicited a response, but one that is noticeably above the quiescent baseline. Critically, both responses started almost immediately after the end of the saccade, indicating that this signal was remapped.

We would note that many studies claim that only information about the attended stimulus is remapped. Yet at the time of a saccade, attention is automatically allocated to the saccade goal (Deubel & Schneider, 1996; Kowler, Anderson, Dosher, & Blaser, 1995), so this standard claim would require that the hypothesized attentional pointer related to a peripherally attended stimulus is also remapped. Remapping of the entire priority map fulfills both of these needs, and thus the remapping seen in LIP is consistent with these claims. A more inclusive hypothesis, which we have previously proposed (Mirpour & Bisley, 2016), is that remapping of the entire priority map might allow more than a single attentional pointer to be involved in securing spatial stability.

## Remapping in LIP: Spatial stability

Our hypothesis on how LIP remapping could be involved in spatial stability relies on three assumptions. First, limited resources in the brain focus on one or more high-priority objects or locations in the visual periphery. This could be similar to the limitation set by visual working memory (Bays, 2018; Bays, Catalao, & Husain, 2009; Luck & Vogel, 1997, 2013), a cognitive process that is known to have a limited capacity. Second, subjects tend to be unaware of objects or locations with low priority, as illustrated by change blindness (Rensink, O'Regan, & Clark, 1997), inattentional blindness (Simons & Chabris, 1999), or mudsplash (O'Regan, Rensink, & Clark, 1999) tasks. This would suggest that specific information from much of the visual field may not be necessary to maintain a percept of stability. The final assumption is that LIP activity acts as a priority map and the activity can be seen as an attentional pointer.

Based on these assumptions, the way remapping could create a percept of stability across saccades is relatively simple. Prior to a saccade, during a period of stable fixation, attentional pointers in LIP identify important objects or locations in the visual scene so that higher-processing areas can access the relevant information from neurons with the relevant receptive fields in earlier visual areas. In the brief period after the eye movement, when the activity in LIP is shifting, the mechanism underlying saccadic suppression limits what information is passed to the higher-processing areas. So by the time the new visual inputs arrive in visual cortical areas from the retina, the attentional pointers have already shifted. This means higher-processing areas gain access to information from new sets of neurons with the relevant receptive fields, as pointed to by the attentional pointers, but the visual information (i.e., the object or location) is the same as it was before the saccade. So from a cognitive perspective, detailed information about the same objects or locations is present both before and after the saccade, and our lack of obvious awareness about the rest of the scene masks other information that might otherwise alert us to the change.

Whether visual stability is driven by the shifting of one, two, or multiple attentional pointers, the finding that activity across all of LIP is remapped in a 25-ms window is consistent with the underlying idea that there is a mechanism in the brain that can take eye movements into account, and this occurs over a temporally constrained window, in which saccadic suppression occurs.

## Characterizing FEF neurons

From a superficial perspective, FEF is often thought be similar to LIP, with the exception that it is closer to the oculomotor plant, as evident by the presence of neurons that respond to learned eye movements made in the dark (Bruce & Goldberg, 1985), the presence of connections directly to relevant brainstem nuclei (Stanton, Goldberg, & Bruce, 1988), and the fact that low-current stimulation can generate short latency eye movements (Robinson & Fuchs, 1969). Like LIP, many of the visual and visual-movement neurons in this area respond preferentially to behaviorally relevant stimuli (Gold & Shadlen, 2000; Thompson et al., 1996), which has been particularly well documented in visual search (Bichot & Schall, 1999, 2002; Cohen, Heitz, Woodman, & Schall, 2009; Cohen et al., 2007; Nelson, Murthy, & Schall, 2016; Sato, Watanabe, Thompson, & Schall, 2003), and it is known to drive attentional modulation in earlier visual areas (Armstrong & Moore, 2007; Moore & Armstrong, 2003; Noudoost & Moore, 2011; Steinmetz & Moore, 2014). As such, FEF has also been described as a priority map (Bisley & Mirpour, 2019), although sometimes the more classical term *salience map* is used (Purcell, Schall, Logan, & Palmeri, 2012; Reppert, Servant, Heitz, & Schall, 2018; Servant, Tillman, Schall, Logan, & Palmeri, 2019). We prefer to avoid the use of this term because it conflates behavioral relevance and low-level salience, yet names the map as if it were primarily driven by low-level sensory attributes. Given that FEF neuronal activity represents priority, one might expect that remapping would play a similar role in FEF as it does in LIP, but as described below, remapping seems to be doing something different in FEF.

To understand the potential role of remapping in FEF, it is necessary to first describe two populations of neurons present in FEF. Neurons in FEF are often categorized based on their responses in the memory or delayed visually guided saccade tasks (Bruce & Goldberg, 1985; Lowe & Schall, 2018) on the assumption that they all play a role in guiding saccades or attention (i.e., that they are part of a priority map), but here we refer to a difference based on behavior. In 2004, Hasegawa and colleagues (Hasegawa, Peterson, & Goldberg, 2004) found a small population of neurons in FEF and in an area anterior to FEF that preferentially responded to a stimulus that the subjects needed to avoid looking at to get a reward. They described these as “don't look” neurons. We recently hypothesized that such a signal could explain inhibitory tagging (Klein, 1988)—the marking of items in visual search that have already been fixated and thus do not need to be examined again—and, using the visual foraging task, we found a subset of about 15% of FEF neurons that preferentially responded to stimuli the subjects had fixated earlier in the trial (Mirpour, Bolandnazar, & Bisley, 2019). Critically, we showed that these neurons, which we will call tagging neurons, had fundamentally different properties from the majority of FEF neurons, which we will call priority map neurons. In the following sections, we look at remapping in these two populations, starting with the priority map neurons.

## Remapping in FEF: Factors affecting future saccades

Although a robust proportion of FEF neurons have been shown to remap when using the traditional metric of response onset, we have been unable to find evidence of stimulus-related response remapping in FEF priority map neurons. It is worth noting that while we tend to think of LIP and FEF as behaving similarly in search (Thomas & Pare, 2007; Thompson et al., 1996), we have found a number of major differences between the responses of LIP neurons and the priority map FEF neurons using the visual foraging task. Perhaps most important, while LIP activity remains robust throughout a trial, FEF activity is often suppressed during extended fixation durations (Mirpour, Bolandnazar, & Bisley, 2018). We have suggested that this suppression is a mechanism for controlling the timing in search: Responses representing items in the visual periphery are suppressed when the animal does not wish to move. That suppression is released a few hundred milliseconds before the saccade, creating a map that ramps up strongly and drives the next saccade. During the leadup to each saccade, FEF activity ramps up strongly at every location representing a stimulus. When looking at the ramp-up response and into the period of suppression following the saccade, we have not been able to find any evidence of predictive remapping of stimulus-related responses (unpublished observations). This does not mean that there is no remapping of stimulus responses in FEF, but if there is, it is minor compared to the clean and consistent remapping seen in LIP.

While we found no evidence of stimulus-related remapping, previous work has shown predictive signals in FEF that represent upcoming behavior. Phillips and Segraves (2010) analyzed the activity of single FEF neurons while animals performed a free-viewing visual search task in which the subjects looked for a small image of a fly embedded in natural scenes. They found two important predictive results. First, the responses of FEF neurons predicted where the upcoming saccade would go starting, on average, 55 ms after fixation onset. For a subset of these neurons, this occurred at or before their visual latency, so the activity representing the saccade goal could not be generated via a feedforward process from the retina. A similar result has since been reported in the superior colliculus in a slightly more constrained form of free-viewing visual search (Shen & Pare, 2014). This suggests that information that will impact the decision about where to look next is already present in FEF by the time the feedforward signal arrives, and this signal is remapped around the time of the saccade. Indeed, Phillips and Segraves's (2010) second key result showed that to be the case: The activity of a specific subset of FEF neurons could be used to predict where the second saccade (i.e., the saccade after the upcoming saccade) would go. This response, which they called advanced predictive activity, started later during the previous fixation and tended to be seen in the same subset of neurons that predictively remapped the goal of the upcoming saccade. The remaining FEF neurons started to represent the goal of the upcoming saccade later in the fixation, and the authors suggest that perhaps these neurons are more likely to generate that saccade, while the other population plans for the next saccade.

More recent work from the same group suggests that this predictive remapping may carry more information than that related to the task (Glaser et al., 2020). Noting that during search, animals have a bias to move away from the edges of the monitor, and the authors found that the remapped activity carried information about this bias, whether the animals ended up acting on it or not. Together with the previous findings, these data suggest that FEF activity carries information about more complex task and non-task-related factors and that these factors are maintained in FEF via the remapping mechanism.

## Remapping in FEF: Keeping track of what has been fixated

**Figure 6.**
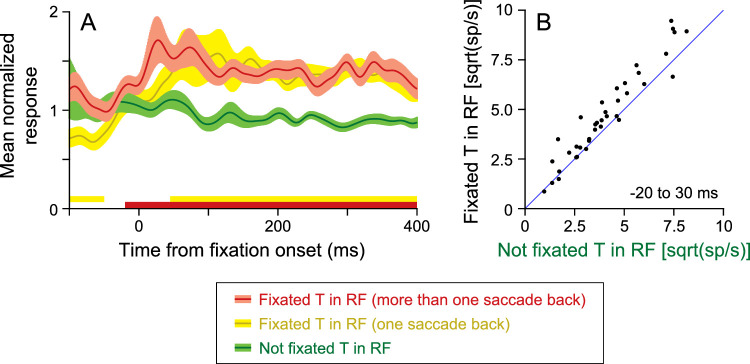
Remapping in tagging neurons in FEF. (A) The mean normalized responses of 28 tagging neurons, aligned by fixation onset, when a T that had not been fixated (green trace) was in the response field (RF), when a T that had just been fixated was in the RF (yellow trace), and when a T that had been fixated earlier in the trial was in the RF (red trace). The width of the traces indicates the SEM across neurons. The lines along the x-axis indicate times at which the red and green traces were significantly different (red line, *p* < 0.05, *t* test) and at which the yellow and green traces were significantly different (yellow line). (B) The responses of 38 putative tracking neurons in a 50-ms window starting 20 ms before fixation onset when a previously fixated T would be brought into the RF are plotted against the response when a T that had not been fixated would be brought into the RF. Modified from Mirpour, Bolandnazar, and Bisley (2019).

As noted above, the small subset of tagging neurons in FEF preferentially responds to stimuli that have been fixated earlier in a trial of visual search. This can be seen by comparing the red and green traces in Figure 6 (Mirpour et al., 2019), which show the response to a target that had been fixated earlier in the trial (red trace, Figure 6A) and to a target that had not yet been fixated and was not the goal of the upcoming saccade (green trace). The thick red line along the x-axis shows the times at which these two traces are significantly different (*p* < 0.05, paired *t* test at each millisecond) and begins approximately 20 ms before fixation onset. In other words, this extra retinal signal is remapped to the postsaccade response field even before the eye stops moving. Indeed, most neurons (21/38; 55%) respond significantly more to a previously fixated target than to an unfixated target in a 50-ms window starting 20 ms before fixation onset (Figure 6B).

While it may be clear why we have remapping of priority in LIP (for perceptual continuity) and of saccade goals in FEF (so that more than one saccade can be planned at once), it may not be so clear why it is needed for this sort of tagging. We proposed that remapping of this tagging signal acts as an efficient form of memory. If inhibitory tagging is a mechanism by which objects on a priority map are represented by lower activity after they have been fixated (Itti & Koch, 2000; Klein, 1988), then one can think of this as a form of working memory. Each tagged object is one that must be remembered. It is thought that explicit working memory has a limited capacity (Bays, 2018; Bays et al., 2009; Luck & Vogel, 1997, 2013), and if this carried over to tagging, then there would be a strong degradation of this map after three or four saccades. Remapping allows the updating of tagged locations across saccades independent of the number of eye movements and does not require a top-down mechanism to individually identify all of the objects that need to be tagged after each saccade. Instead, remapping shifts the current map after each saccade.

On the basis of this mechanism, one could imagine that the tagging signal could be brought onto the map by remapping the responses of fixation neurons: the neurons in FEF that respond during periods of intentional fixation (Bizzi, 1968; Bon & Lucchetti, 1990; Izawa, Suzuki, & Shinoda, 2009; Suzuki & Azuma, 1977). In this way, the brain could automatically keep track of everything that has been fixated. However, the tagging response of the stimulus that had just been fixated (yellow trace in Figure 6A) did not remap with the rest of the map but began 50 ms after fixation onset (see yellow line along x-axis). This suggests that the tagging signal first appears via a different, likely top-down, input and then gets remapped once it is in this network. Indeed, this would explain why inhibitory tagging is only present when subjects search for an object (Dodd, Van der Stigchel, & Hollingworth, 2009): If there is no need to remember where you have looked, then you do not have to tag that object on the map.

## Conclusion

In the past 30 years, it has become clear that neurons in a number of cortical and subcortical areas remap to take into account shifts in gaze due to saccades, and many have used these data to suggest this as a neural mechanism for spatial stability. We have suggested that to best understand the role of neural remapping in natural behavior, one must study it with tasks that mimic natural viewing conditions. Using the results of such tasks, we propose that remapping in LIP could play a role in spatial stability by shifting attentional pointers during a brief window immediately after a saccade, during which perception is still suppressed. We have also proposed that remapping in FEF plays two roles, depending on the neuronal population. In priority map neurons, remapping appears to be involved in updating saccade goals when planning more than one saccade at a time. And in tagging neurons, remapping allows subjects to keep track of which items have been fixated during search without being limited by the limitations of short-term memory capacity. These roles show the multifaceted benefits of a mechanism that can account for shifts in gaze and highlight the importance using naturalistic behavioral tasks.
